# Development and Usability Testing of an Internet Intervention to Increase Physical Activity in Overweight Adolescents

**DOI:** 10.2196/resprot.2410

**Published:** 2013-01-28

**Authors:** Kirsti Riiser, Knut Løndal, Yngvar Ommundsen, Turid Sundar, Sølvi Helseth

**Affiliations:** ^1^Institute of NursingFaculty of HealthOslo and Akershus University College of Applied SciencesOsloNorway; ^2^Department of Coaching and PsychologyNorwegian School of Sport SciencesOsloNorway; ^3^Department of Primary and Secondary Teacher EducationFaculty of Education and International StudiesOslo and Akershus University College of Applied SciencesOsloNorway; ^4^Diakonova University CollegeOsloNorway

**Keywords:** Internet, intervention, development, usability testing, adolescents, physical activity, overweight

## Abstract

**Background:**

Internet interventions may provide opportunities for low threshold counseling using feedback to guide and support health behavior, including increased physical activity. Research shows that overweight and obese adolescents are less physically active than their peers of normal weight. There are good reasons to believe that Internet-based interventions may be particularly suitable for motivating adolescents to increase physical activity, but we need to gain further knowledge of what features are effective and how to design such interventions.

**Objective:**

To describe the process of development and evaluation of usability of a Web-based program for increasing physical activity in overweight adolescents.

**Methods:**

Informed by the self-determination theory, motivational interviewing, and perspectives on self-regulation, this intervention was developed in a stepwise process by an interdisciplinary team of researchers, designers, developers, and representatives from the target group. An iterative qualitative usability testing approach (observation, survey, and interview) was applied in 2 sequences, first in the lab and second in the field, to assess how adolescents (aged 12-16 years) used and experienced the program and to make adjustments to the program based on evaluation of their response.

**Results:**

The following components were included in the program: self-monitoring through planning and registration of physical activity and graphical response on progress, autonomy supportive individual Web-based counseling, forum for social support, and relevant age-adjusted information about physical activity. The first usability test resulted in adjustments related mainly to making the content and aim of the different features more visible and explicit. The second test evaluated the program with adjustments from the first test, revealing that the program was well accepted by the participants and only small aesthetic adjustments had to be made to complete the final version of the Internet program, Young & Active.

**Conclusions:**

Thorough preparation, with clear theory foundation and close monitoring in the developmental phase, as well as contribution and iterative evaluation from the target group, is essential to create a user-friendly and engaging program. The efficacy of the program will be evaluated in a controlled trial.

## Introduction

### Background

Investigations point to a decrease in physical activity (PA) from childhood to adolescence [1]. Adolescents who are overweight with obesity are even less active compared to peers of normal weight [2]. Research also shows impairment in health-related quality of life among overweight and obese children and adolescents [3]. Aerobic fitness is positively associated with physical and mental health independent of body mass index, and a substantial weight reduction and maintenance over time may be unachievable for many overweight individuals [4,5]. Thus, there are good arguments for interventions to emphasize increased habitual physical activity more than individual weight regulation. It is also essential to focus on interventions to increase and maintain PA in adolescence because of the relations between activity patterns in adolescence and adulthood [6,7].

Recent research suggests that Internet technology is a promising way to change a person’s health behavior and therefore provide opportunities for low threshold counseling using feedback to guide and support health behavior [8]. It might seem paradoxical to use an inactive device to promote activity among adolescents. However there are good reasons to believe that Internet interventions for promoting PA may be particularly suitable for this age group. Digital media is an area in which adolescents are experts and technology is the means of their empowerment [9]. Instead of thinking of digital media solely as the cause of physical inactivity, becoming overweight, and obesity, we can choose to use adolescents’ competence as a valuable basis for raising the efficiency of communication in health promotion efforts. Despite promising results of Web-based interventions to promote PA in children and adolescents, well designed research is needed to further enhance our understanding of intervention characteristics that best promote behavior change [10-12].

An Internet program called Young & Active was developed aimed at motivating overweight adolescents (aged 13-14) to increase and maintain PA and thereby enhance their fitness and health-related quality of life. The intervention study is built upon the framework for researching complex interventions given by the Medical Research Council [13]. This framework is developed for the purpose of evaluating interventions in natural or everyday practice. The development and evaluation process is divided in 4 phases: (1) development—identifying and developing the evidence base, theory, modeling processes, and outcomes, (2) feasibility and piloting—testing procedures, estimating recruitment and retention, and determining sample size, (3) evaluation—assessing effectiveness, understanding change process, and assessing cost effectiveness, and (4) implementation—dissemination, surveillance and monitoring, and long term follow-up. This article describes the process and result of the development and how theory informed Young & Active with a particular focus on how the end users, the adolescents, through development and usability testing, contributed to the final version of the program (Phase 1). The development and usability testing will be followed by a 12 week controlled trial (Pilot-Phase 2), and finally by a full scale RCT (Phase 3).

### Theoretical Basis and Content of Young & Active

Extensive use of theory and inclusion of behavior change techniques is important in Internet interventions to increase effectiveness [8]. The present intervention aimed to stimulate adolescents to engage in self-chosen activities that they find fun, meaningful, and want to do. Research shows that individuals who have more autonomous reasons for exercising are more positive toward PA [14] and more likely to initiate and maintain PA [15]. Fun, enjoyment, social support, and to a lesser extent health benefits, are reported as predictors of participation in PA. Especially for girls, the activity must be on their own terms [16]. Self-determination theory (SDT) has proven useful in understanding motivational, cognitive, and affective processes of physical activity [15-17]. Therefore, it was chosen as the theoretical framework for this intervention, supplemented by aspects of self-regulation theory [18,19]. Central to SDT is the question of how people internalize and integrate extrinsic motivations and come to self-regulate their behaviors in order to engage autonomously in their daily life [20]. According to this theory, developing a sense of autonomy and competence as well as relatedness is essential to make a person more self-regulated and able to sustain the behavior [21,22]. Autonomy reflects the need to engage in activities with a sense of choice, competence represents the feeling that one can accomplish tasks and reach goals, and relatedness refers to the sense of being understood and respected by significant others [21,23]. SDT supplemented by perspectives on self-regulation of behavior change gives suggestions on how an autonomy-supportive counseling style can motivate people to change health behavior, in this case, to increase physical activity [24]. Autonomy support, structure, and intrapersonal involvement are the 3 dimensions of the social environment that can support the need for autonomy, competence, and relatedness. If these factors are presented in an autonomy supportive manner [25], they can facilitate physical activity adoption and maintenance [26]. By giving the adolescents opportunity to form goals for PA, make a plan for how to reach these goals and monitor them, the program might facilitate autonomy. Individualized autonomy supportive feedback from a counselor based on the adolescents’ registrations in the program is believed to provide users with a sense of autonomy, competence, and relatedness. It is also shown that SDT can offer a theoretical rationale for understanding the efficacy of motivational interviewing (MI), a client centered counseling method to promote behavior change [27]. MI involves avoiding controlling behaviors and direct persuasion. By expressing empathy, making the participant more aware of discrepancies between goals and actions, encouraging personal reasons for change, and supporting self-efficacy, the MI approach seeks to empower the participants’ own reasons for change [27]. Thus, theoretical inputs from SDT and principles from MI are used in the counseling of the adolescents to promote behavior change in this intervention. Table 1 presents this in a schematic form. We aimed to develop a need-supportive program which hopefully will make the adolescents experience support in a way that enhances their self-regulation and autonomous motivation to increase and maintain PA.

**Table 1:** Relations between motivational styles from SDT and MI—how strategies can be applied to facilitate autonomous motivation to increase and maintain physical activity.^a^

Principles from autonomy supportive counselling:	Principles from MI:	Examples of practical use:
Support autonomy	Avoid coercion and pressure	Do not pressure or argue the case for change (eg, “you have to be more active and exercise more”).
	Explore the adolescent’s own reasons for change	Let the adolescent explore his/her own reasons for being active and exercising.
	Encourage change talk	Affirm and reinforce expressions of problem recognition, exercise, desire, and intention to change.
	Explore options	Let the adolescent choose his or her preferred courses of action (ie, how and when to exercise).
Provide structure	Develop goals	Help to set goals for PA and exercise. Make sure the goals are appropriate, realistic, and achievable.
	Give clear information	Give information about what to expect from exercising and what it takes to achieve self-determined goals. Make sure the information is neutral, clear (understandable), sufficient, and repeated.
	Offer advice	Offer advice when appropriate, but avoid imperatives (eg, “you must exercise regularly”).
	Provide feedback	Follow up goals and plans with regular feedback. Ensure that the feedback is received and understandable.
	Support self-efficacy	Make sure to affirm effort, success, and progress.
Be involved	Express empathy	Display interest in the adolescent and his/her well-being.
	Explore concerns	Reassure the adolescent that their concerns are natural. Acknowledge and explore worries.
	Demonstrate understanding	Try to see the adolescents’ point of view.
	Avoid judgement	Do not blame or criticise the adolescent (eg, “you have failed in following your plan this week”).

^a^ adapted from [27,28]

Considering the age of the target group, we wanted to develop a program with self-explanatory and time-efficient functions [29]. This included a system that could, based on online registrations by the participants, calculate the accurate amount of self-reported low-, moderate-, and high-intensity activities related to time spent, and to make graphic presentations showing progress. Activities relevant for Norwegian adolescents were adapted from the Compendium of Energy Expenditure for Youth [30]. The compendium provides a classification system that standardizes the metabolic equivalent of task (MET) intensities of physical activities used in research. Based on categorization as light (< 3 METs), moderate (3-6 METs) or vigorous (> 6 METs) intensity activities [31,32], we coded activities as green, blue, or red, respectively. Thus, total amount of activity of different intensities would be easily identified in a graphic presentation of bar charts. Self-monitoring is central to the process of self-regulation [18,19], and is shown to increase effectiveness in interventions designed to promote healthy eating and physical activity [33]. It was expected that monitoring of progress and getting autonomy-supportive feedback emphasizing progress will enhance the Young & Active participants’ perception of competence and make them more conscious about how active they are and how increasing all kinds of PA throughout the day can contribute to a total enhancement of activity. Supplied with the narratives, such registrations will provide the counselor with broad information on the participants’ process.

The intervention includes daily registration and narratives on PA by the adolescents along with weekly individual Web counseling from trained health counselors. The initial components of Young & Active and their main content are outlined in Table 2. Except for one initial face-to-face meeting with a counselor to map PA, to be introduced to the program and be assisted in the making of the first PA goals and plan, all contact between participant and counselor will be online. The choices of components for the intervention were supported in a systematic review of Internet-delivered health interventions [34]. The review points to peer support, counselor support, email/phone contact, and updates of the website as intervention characteristics related to better exposure. Internet interventions aimed at adolescents or young adults seem to be most effective when they include combinations of several strategies [35].

**Table 2:** Overview of planned content of Young & Active.

**Mapping of physical activity**	
	Face-to-face interview between counselor and participant based on principles of MI and autonomy supportive counseling (ASC) from SDT, to help the participant reflect on PA, map current level of PA, and outline the possibilities for change.
**Goals and plan for physical activity**	
	Instruction on how to fill in (preliminary) goals and a plan for PA during the day and week. Focus on the value of self-determined goals and activities.
**Registration of daily physical activity**	
	Registration of activities during the day (time of day, type of activity, time spent, and alone/together with someone else).
**Narratives of PA experiences during the day**	
	Free text comments on experiences, feelings, and thoughts on physical activity and exercise and on life in general.
**Automatic feedback on progress**	
	Graphic feedback displaying planned and registered activity for present week and past weeks participating in the program.
**Tailored feedback from counselor**	
	Weekly individual written response from the counselor based on the participant’s goals, plan, logs, and diary notes. Feedback based on principles from ASC and MI.
**Evaluation and adjustments of goals and plan**	
	Encouragement via tailored feedback to regularly evaluate and adjust goals and plan in accordance with progress.
**Forum**	
	User-driven forum for support and the sharing of physical activity related experiences and tips.
**Information on physical activity**	
	Relevant information on PA and sports activities. Regularly updated and edited.

### Usability Testing

Young & Active focuses on how adolescents can find their own source of motivation for increasing and maintaining PA. It is proven that the intervention in itself is motivating to use in the sense that the end users choose to visit, use, and revisit it [29]. We aimed for the intervention to be appealing, to include intuitive functions, to be easy to navigate, and provide understandable and meaningful information. Testing by the end users—the adolescents—is necessary to ensure that these components are met. Usability testing refers to evaluation through the analysis of typical end users interacting with the program, allowing for iterative modifications [36]. Qualitative feedback through testing with representatives from the target group can give valuable information on user experience and help developers determine if a program will turn out to be effective and achieve its purpose [37,38]. The vast amount of usability problems and issues can be identified with only a small number of test subjects, as few as 8 to 10 participants [36]. A cycle of design-evaluation-redesign has the potential of major reduction in usability problems [39], which makes usability testing a powerful method for evaluation before inclusion in a trial.

## Method

### Development of the Program

**Figure 1:**
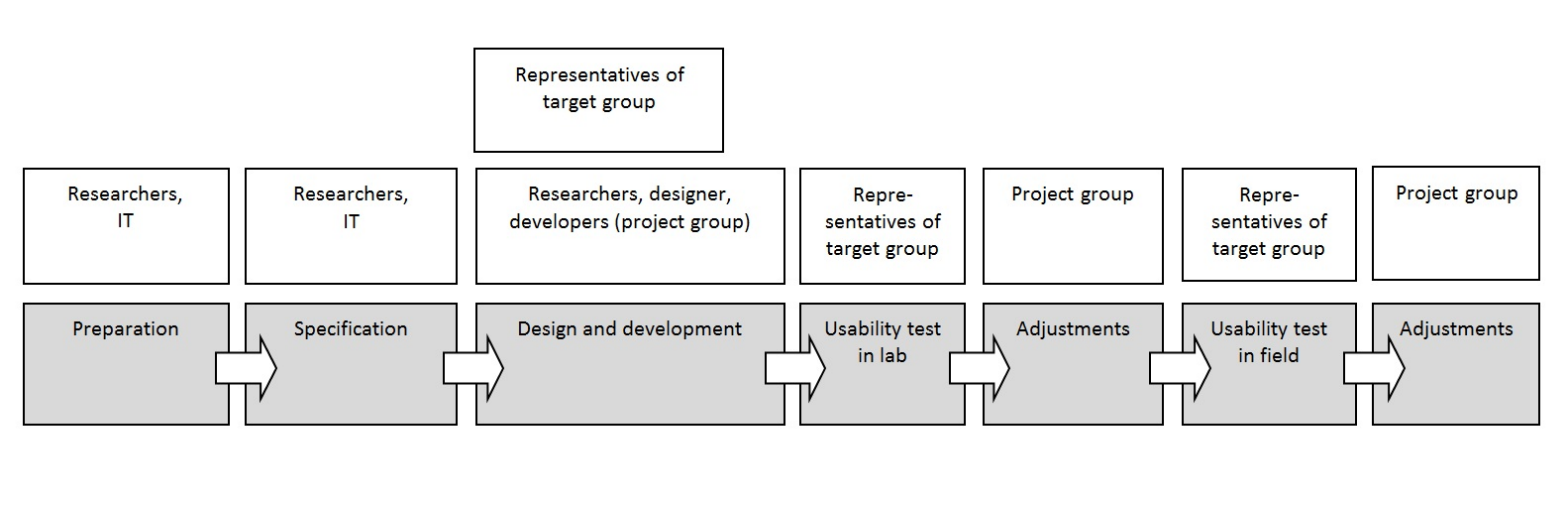
Development process of Young & Active.

The process of development of Young & Active covered the following steps, including preparation, specification, development, usability testing in 2 different settings, evaluation, and adjustments (Figure 1).

The preparation step included choice of theoretical framework and mapping of user needs related to physical activity and use of Internet. Risk analyses were made with assistance from the Department of Information Technology at Oslo and Akershus University College of Applied Sciences to assure that potential threats to the anonymity of the adolescents were accounted for. A detailed specification of requirements for the program was developed. An interdisciplinary team of health and education researchers, graphical designers, and application developers formed the project group.

Based on risk analyses and specification of requirements, the outlined content was transformed into wireframes (a visual guide that represents the skeletal framework of a website) to visualize and illustrate the link between text and graphics and interactive features. Development started with ideas being picked from well-established websites for adolescents with functions such as forums, ask-the-expert, and information on sexuality, health, and adolescence in general for instance. Design, language, use of symbols, and site architecture were studied. Representatives from the target group of adolescents (n=4), strategically sampled from an ongoing project for obese youth, participated in a workshop to help decide on design, content, and functionalities. The workshop was an informal setting with open discussions on the presented wireframes. Comments and suggestions from the adolescents were noted and included in the ongoing developmental process when found relevant.

### Testing Usability in Lab and in Field

An iterative qualitative usability testing approach with observation, a questionnaire, and focus group interview was used to assess how the participants use and experience the intervention. Usability tests were carried out in 2 sequences, first in a lab setting and second in the field, over a period of 2 weeks. The Honeycomb model [40] served as a guideline for the tests. According to this model, the quality of experience of a program is dependent on whether the users find the program valuable, useful, usable, desirable, accessible, findable, and credible. The tasks and questions for the questionnaire and interviews were grounded in these facets to ensure that they were all focused in the different components of the program. The questionnaire included questions that were answered Yes/No/I don’t know, and also the participants were encouraged to write suggestions for changing and improving the program. The focus group interviews allowed the adolescents to speak more freely about their impression of Young & Active.

As mentioned, the aim of the tests in lab and in field was to assess how adolescents in the target group used and experienced the prepared intervention. For this reason, strategic sampling was conducted among adolescents who were expected to give valuable information. The number of participants was decided upon according to an appraisal of achieved saturation of qualitative information about usability [41]. In order to strengthen the trustworthiness of the information, different types of qualitative material were gathered in the field through triangulation by using several research methods [42].

The first test (lab) was carried out in 2 groups of a total of 7 adolescents (aged 12-13 years), recruited from a school and a project for obese adolescents respectively, and took about 90 minutes per group. At this point, the program contained all the main functions except to the forum and feedback page. The participants initially received brief information about Young & Active. Listed tasks then guided them through the different parts of the program. Each participant was observed by one of the members of the project group. Following the practical tasks, the adolescents filled in the questionnaires. Finally, focus group interviews were conducted in both groups.

To perform technical evaluation, to assess how the users respond to using the program over time, and to get a more thorough evaluation after adjustments based on the first usability test, the Young & Active program was tested over a period of 2 weeks. From an exercise group for overweight and obese youths, 8 adolescents (aged 14-16 years) were recruited. Informed consent to participate was given by the adolescents and their parents. The participants first met with the researchers for an informal conversation focusing on feelings, thoughts, and experiences with different forms of PA. The conversation was informed by the MI approach [27] and was aimed at making the adolescents reflect on goals to increase and maintain PA. The participants received a personal user identifier and a password, and were given a brief introduction to the different functions of the program. If desired, they received help to form goals for PA and to set up an activity plan to reach these goals. The adolescents started registrations the following week after getting a reminder via SMS. The counselors gave individual feedback twice during the test period. Bugs and minor errors were reported by a message in the program or via the forum, and consecutively handled by the programmer. The researchers moderated the forum and message system. An extended version of the user experience from the first test and focus group interview followed the week 2 test. Registration of use (frequent registrations, diary notes, and posts in forum), were summarized.

### Data Analysis

The combination of questionnaires, observations, and focus group interviews was used to triangulate data to control and support the different findings, as the discussions in the focus groups were used to broaden and support the outcomes of observation and questionnaires. After each of the 2 tests, data from the questionnaires were summarized. Answers from the open-ended questions from the questionnaire and notes from observations and interviews were analyzed as text, using simple content analysis [43]. These data were sorted to correspond with each of the different pages of the program (My page, activity plan, diary, feedback, forum, and info page). General categories like user performance, visual design, content, functionality, and motivation for use were identified.

## Results

### Results From the Process of Development

**Figure 2:**
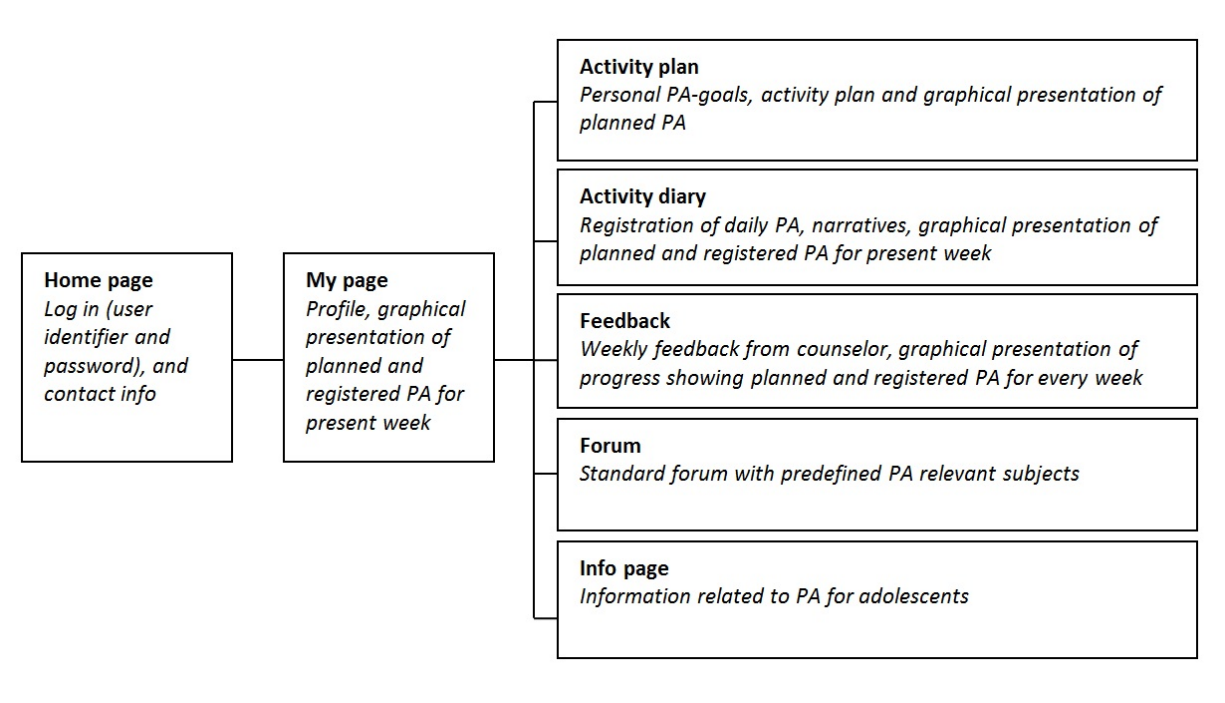
Main content and interactive features of Young & Active.

The first part of the development process resulted in a draft with suggestions on name of the program, logo, color scheme, and page layout, including the content and placement of different functions distributed. A flow diagram of the Young & Active program as it was initially planned, showing the main content, interactive features and the links between the different pages, is provided in Figure 2.

No changes were made to this structure during development, and the pages described in Figure 2 are all represented in the final version of Young & Active. In addition, we designed a simple password-protected page for counselors. On this page, counselors can view a summary of each participant’s registrations and diary notes, write and send weekly feedback, and monitor the forum. Young & Active was developed in eZpublish (version 4.4.0), based on Open Source technology and designed to support PCs, Macs, and newer versions of all browsers. Usability was not tested for mobile devices, but the program shows acceptable readability for smartphones and tablets.

### Response From Workshop

The workshop participants liked the suggested name of the program. Colors and graphics, especially the smiley logo, met with approval. The participants focused on the advantages of using smileys in the communication as a way of making it easier to express feelings and the communication more effective. The benefits of written self-determined goals and detailed plans for activity were emphasized, but the adolescents did not immediately understand what kind of information the PA-registration page required. It was obvious that thorough explanation and specific examples were necessary to get a quick understanding of the different functions. The participants saw the advantages of bonding with other adolescents in the program to exchange experiences and opinions and to ask questions. On the info page, the adolescents indicated they would prefer to read about activity alternatives and how to make healthy food. Feedback on the wireframes was mainly positive. Suggestions on changes and supplements to graphics, features, content, and functionality from the workshop were incorporated in further development of the program.

### Results From Usability Tests in Lab and in Field

The participants of both lab and field tests had a computer at home and regarded themselves as competent computer users. All of the participants in the field test used Young & Active at home in one way or another during the test period, meaning that they made at least 1 registration in the diary, posted in the forum, or sent a message to the counselors via the message system. Most of the participants (5/8, 63%) made regular, frequent registrations and used the diary, while 1 participant got ill, 1 had an ankle injury and 1 responded “this program is not for me, but I am sure others will find it useful”. This last participant joined in on the survey and interview, while the other 2 infrequent users did not.

#### User Performance

The participants in the lab test had no problems logging in or navigating among pages. They tended to ask for help rather than using help buttons with explanatory text. All participants found relevant leisure sports activities, but did not intuitively choose activities for the entire day and week (to and from school, at school and so on). This occurred both when planning and making registrations in the diary. In the plan, the adolescents found it inconvenient to have to select days one by one for activities performed every weekday, such as walking to school. Searching through lists of activities in the plan and the diary were shown to be time consuming.

The most important adjustments after the first usability test concerned making the content and the aim of the different features more visible to the users. This included choosing bold headings, making short introduction texts for every task and creating more visible help-buttons with pop-up texts for specific explanations and tips.

Observation during introduction of the program and the 2-week test period revealed that the participants in the second usability test very quickly grasped the different functions of the program. Due to the short test period, none of the adolescents made changes in their activity plan, but 2 participants supplemented their goals for activity. The 5 active users made registrations according to their activity plan and added other less regular activities if relevant. They estimated registration to take less than 5 minutes. All active users wrote in the diary, some almost every day and others less frequently. Except for minor bugs, no technical problems occurred that compromised use of the program in the field.

#### Visual Design

Visual design refers to the informants’ impressions of how the program looks and how they find the colors, fonts, illustrations, and layout. Both groups in the lab test approved of the color scheme, the program smiley logo that has not been seen by some participants before, the design in general, and found it appropriate for both boys and girls. Participants in the second usability test also approved of the design and graphics and the smileys in particular. However, they had complaints on both visual and functional design of the forum. It did not appeal to them aesthetically, as the text fields were too spacious and the profile smileys too large.

#### Content

All in all, the participants in the lab test commented positively on the content of the program. They appreciated the opportunity to plan and log activity, but were mainly concerned with goals, plans and registration of sports activities and exercise, not PA in general. Some suggested pop-up text with tips on what to write about in the diary. The adolescents easily made goals for PA, although some were unsure about how specific the goals had to be and thus asked for help. The participants reported liking the info page, noting that it was understandable and the issues were relevant, but several of the informants asked for more facts about effects of exercise and more pictures and videos to illustrate the information.

No significant changes had to be made to the content after the lab test, other than adding pop-up boxes with tips, more pictures, and more fact-oriented information on the info page. Some requests were rejected due to security demands (personal profile) and time and budgetary constraints (exercise videos).

The adolescents participating in the usability test in field had no problems making goals, plans, and registering in the diary, but asked for more options for activities during school time (eg, field trips). They appreciated the opportunity to reflect on their daily activities and the day in general, but requested a less structured diary with fewer leading questions and 1 single text field with an open question (“How was your day?”) instead of 3 fields with more specific questions. The info pages, both text and illustrations, were reported to be fine. Feedback from counselors was valued (“It motivated me!”*)*, but could be given more often. All in all, the informants found the content of the program interesting and uncomplicated.

#### Functionality

Functionality refers to the interactive and adaptive features of the program. The participants appreciated the possibility of making a profile. Graphs and the fact that they interactively responded to changes in registrations got positive remarks. After the lab test, the project group decided on an additional feature to give the participant and counselor an opportunity to exchange short messages independently of the diary/feedback if necessary, and included a mailing system with a message box present on every page. This was mainly for security and practical reasons.

The adolescents in the second test found it easy and fun to keep track of amount and type of activities by observing the bar charts over time. However, 2 informants reported that the bars showed variable stability depending on the browser. The forum got the most negative feedback as the adolescents experienced it as “not user friendly” and indicated that it was “hard to get an overview”. Response to this was given both as messages in the forum during the test period and in the interviews that followed. The interviews revealed that not all of the adolescents had noticed that they had received feedback from the counselor and that this had to be more explicitly announced on My page.

#### Motivation for Use

All of the participants in the lab test focused on the importance of making goals and a plan to increase PA, commenting that, “it is easier to hold on to goals that I have written”. However, they were not convinced that they would make an effort to register and write in the diary every day, and they requested the possibility to backdate. Most participants (6/7, 86%) reported that Young & Active might contribute to making them more active. Based on this response we made the operations for registering activities more intuitive and time efficient, and made it possible to backdate within the same week so registrations did not have to be made every day.

In the field test, the youngest adolescents (age 14) were most positive regarding how useful and interesting the program was, giving comments like, “it made me more conscious about PA”, “the program proves that I am more active than I think I am”, “I liked the program, registrations are fun, I would like to continue to use it”. The older adolescents (age 16) were more uncertain, commenting that, “it takes some time and it is hard to remember to register, so I am not sure if I would have liked to use the program over time”.

### Results of Usability Test in the Field, the Finalized Young & Active Program

Only minor adjustments were made after the usability test in the field. These included better marking of feedback from the counselor, less spacious commentary fields in the forum, and 1 instead of 3 text fields and questions in the diary. Procedures for feedback once a week remained, but an initial personalized message by the message system was included in the intervention procedures. We also picked up responses from the preliminary workshop and positive feedback in the tests regarding the smileys, and added a function for choosing a smiley to supplement the narratives in the diary for expressing the “mood of the day”. Technical adjustments and subsequent tests were made to reassure stability independent of browser.

**Figure 3:**
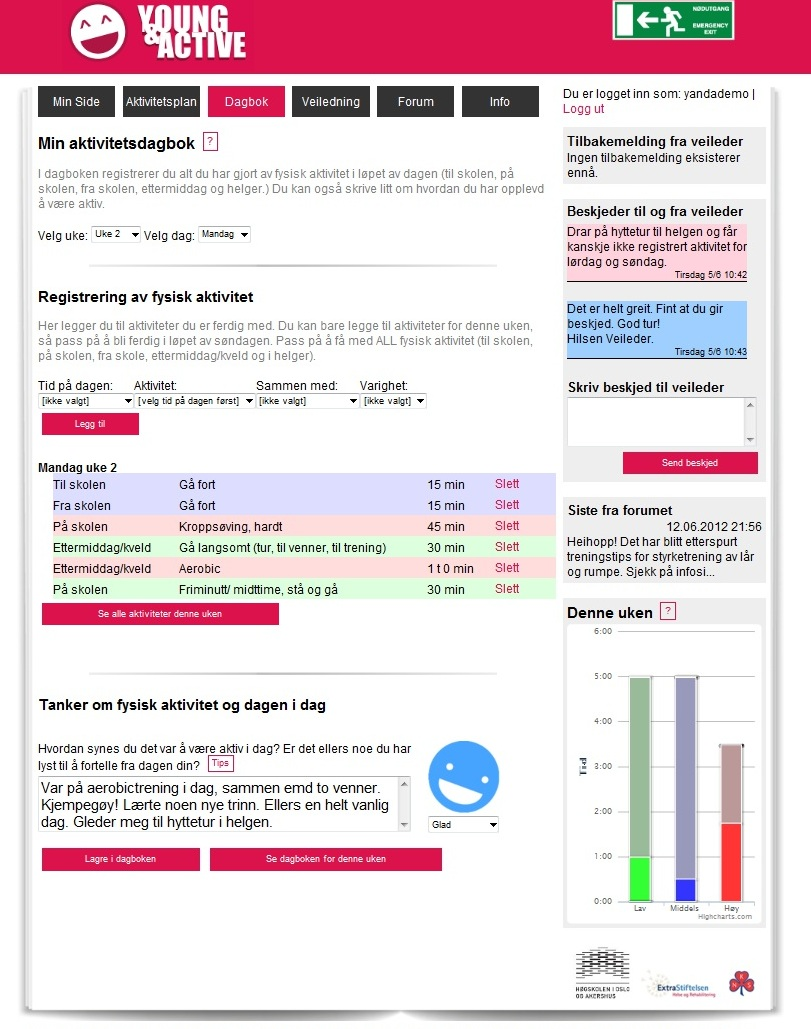
A screenshot of the page for daily registration and diary notes on physical activity. The right panel has links to the last feedback from counselor, short messages to and from counsellor, the last comment on the forum, and a graphic presentation of the planned and registered activity for the present week.

The end result of the development and usability testing was a program that included the components as described, but with cleaner design, clearer instructions, and more intuitive and time-efficient functions. See Figure 3 for an example of one of the program pages.

## Discussion

This article describes the development and results of usability testing of Young & Active, an Internet intervention specifically designed to increase PA in overweight adolescents. A sample of representatives from the target group participated in the development and usability testing, and gave valuable suggestions regarding design, content, and functionality. Accessing the intervention website and actually using it is essential for an Internet intervention to succeed, that is, to induce behavior change [35]. To choose to stay online, engage in the intervention, and to revisit it to follow up and complete the different tasks is definitive to whether the intervention works or not. Only the adolescents themselves can express their preferences for a program like this, thus, they are valuable creative partners in the developmental process. Inclusion of end users in the making and formative evaluation of the program is also in accordance with the chosen theoretical framework in that the adolescents’ perspective and competence is acknowledged [22].

Adolescents are considered familiar with many online programs and are well aware of their preferences regarding layout, graphic appearance, colors, illustrations, fonts, and so on. Feedback from the workshop and usability tests reassured us that the adolescents liked the design, and particularly the use of specially designed smileys, which originated from well-known emoticons (emotion icons), in the logo and profile. Such emoticons can be regarded as non-verbal cues and are considered a creative and salient way to add expression to strict text [44,45]. Although they were not included in the program by the time of the usability tests, we assumed that supplementing the diary with optional smileys might help the adolescents communicate about feelings toward PA, exercise and their day in general.

The workshop indicated that explicit descriptions, clear intentions, and logic procedures of the different functions of the program are most important. In spite of this being focused in the following development, the first usability test still uncovered challenges with the understanding of the function of the different tasks (ie, making plan and registrations). Literature on how to write for Web emphasizes the importance of making short, concise texts with meaningful sub-headings and one idea per paragraph [46]. Use of pop-up boxes with supplemental text made it possible to reduce static text on the page. Face-to-face introductory instructions and a short session of training prior to getting started assured that the participants in the field test understood the program, its tasks, and its functions.

In addition to appreciation and understanding of the practical use of the program, it is essential that the adolescents find the program useful and that they acknowledge and value the aim of the intervention [40], which is to increase and maintain self-chosen PA that they find meaningful and want to do. The workshop and the first usability test revealed that the adolescents tended to equate PA with exercise and sports. This is unfortunate for at least 2 reasons. First, there is some evidence that, compared with those of normal weight, overweight adolescents participate less in sports and have less positive feelings towards PA [47]. The Young & Active program introduces the adolescents to a broader meaning of PA, comprising, for example, playing with the dog, biking to school, walking about with friends, and playing soccer in the yard. This might make it easier to find and take part in feasible activities without prior negative associations. Second, meeting the recommendations of a total of 60 minutes of daily activity of at least moderate intensity (3 MET or more) [48] is unrealistic for most adolescents when solely including exercise or organized sports. Our intervention thus focuses strongly on the profits of engagement in all kinds of PA (in-school and leisure-time), not just planned and structured exercise, but also shorter bouts of PA. In the final version of Young & Active this message is included in the preliminary mapping interview (verbally), in the written instructional texts, and in the weekly feedback. In addition, the system for registration supports this by summarizing and visualizing the amount of all kinds of PA produced. Participants in the field test reported being motivated by the potential of increasing daily activity. The fact that they found changes in the bar charts rewarding gives hope to our goal of making adolescents more conscious and positive about daily PA when making specific goals, plans, registering, and monitoring the activity [19].

Computer-based interventions have several advantages. These include the benefits of standardization, tailoring, data collection, testing of theory, and practical use [23]. Standardization of the intervention ensures that the content is delivered equally to all participants. Young & Active also provides the flexibility of tailored automated feedback and need-supportive counseling based on registrations by the participants. As discussed, opportunity for daily PA recordings has potential benefits for the adolescents. Additionally, such recordings provide rich quantitative and qualitative data for the investigators. Another important benefit is the potential for reaching out to adolescents who might find it difficult to disclose sensitive information regarding their own health practices face-to-face with an adult. Computerized interventions also have the potential for increased cost-effectiveness compared to more time-consuming direct interventions.

More extensive use of theory has been associated with larger effect sizes [8], however, so far there is a lack of theory-driven interventions with the potential of explaining mechanisms of physical activity behavior change [23]. Young & Active represents an attempt to develop a such a theory-based program for the promotion and maintenance of PA in overweight adolescents. The ongoing study will assess the extent to which use of SDT as framework and the chosen modes of delivery might impact the efficacy of the intervention in the way that the participants increase their fitness and health-related quality of life.

The potentially biggest disadvantage for an Internet intervention such as Young & Active is its limited lifetime. The speed of development of Internet-based programs is vast and there is reason to believe that adolescent users are not particularly faithful in that their preferences shift rapidly. Considering the time-consuming process of development and testing in a research setting, it is a challenge to create a program that has not outlived itself before meeting its audience. Through development and usability testing of the program, we have taken into account the preferences of the target group and hope that this ensured a program that is relevant for the time being. Technology is constantly developing, and new innovative applications appear, which in time will threaten to outdate the design and functions of Young & Active. Nevertheless, the ongoing study will add to the testing of our theoretically informed, individualized, computerized intervention and if the chosen characteristics are suited to promote behavior change.

This study highlights the importance of thorough preparation with explicit theory foundation in the developmental phase and iterative usability testing throughout program development. Most important is the engagement from a sample population for ensuring that the users like, understand, and value the program. Integrating such feedback from the target group is highly valuable in the developmental process and increases the chances of making a potentially effective program. The final usability test showed that the program was well accepted by the participants and can be considered ready for further evaluation in a controlled trial.

## Abbreviations

ASC: autonomy supportive counseling
MET: metabolic equivalent of task
MI: motivational interviewing
PA: physical activity
SDT: self-determination theory

## References

[1] Riddoch CJ, Bo Andersen L, Wedderkopp N, Harro M, Klasson-Heggebø L, Sardinha LB, Cooper AR, Ekelund U (2004). Physical activity levels and patterns of 9- and 15-yr-old European children. Med Sci Sports Exerc.

[2] Olds TS, Ferrar KE, Schranz NK, Maher CA (2011). Obese adolescents are less active than their normal-weight peers, but wherein lies the difference?. J Adolesc Health.

[3] Wille N, Bullinger M, Holl R, Hoffmeister U, Mann R, Goldapp C, Reinehr T, Westenhöfer J, Egmond-Froehlich A, Ravens-Sieberer U (2010). Health-related quality of life in overweight and obese youths: results of a multicenter study. Health Qual Life Outcomes.

[4] Blair SN, Brodney S (1999). Effects of physical inactivity and obesity on morbidity and mortality: current evidence and research issues. Med Sci Sports Exerc.

[5] Fogelholm M (2010). Physical activity, fitness and fatness: relations to mortality, morbidity and disease risk factors. A systematic review. Obes Rev.

[6] Telama R, Yang X, Viikari J, Välimäki I, Wanne O, Raitakari O (2005). Physical activity from childhood to adulthood: a 21-year tracking study. Am J Prev Med.

[7] Leslie E, Fotheringham MJ, Owen N, Bauman A (2001). Age-related differences in physical activity levels of young adults. Med Sci Sports Exerc.

[8] Webb TL, Joseph J, Yardley L, Michie S (2010). Using the internet to promote health behavior change: a systematic review and meta-analysis of the impact of theoretical basis, use of behavior change techniques, and mode of delivery on efficacy. J Med Internet Res.

[9] Buckingham D (2008). Introducing identity. Youth, identity, and digital media.

[10] Nguyen B, Kornman KP, Baur LA (2011). A review of electronic interventions for prevention and treatment of overweight and obesity in young people. Obes Rev.

[11] Hamel LM, Robbins LB, Wilbur J (2011). Computer- and web-based interventions to increase preadolescent and adolescent physical activity: a systematic review. J Adv Nurs.

[12] Lau PW, Lau EY, Wong del P, Ransdell L (2011). A systematic review of information and communication technology-based interventions for promoting physical activity behavior change in children and adolescents. J Med Internet Res.

[13] Craig P, Dieppe P, Macintyre S, Michie S, Nazareth I, Petticrew M, Medical Research Council Guidance (2008). Developing and evaluating complex interventions: the new Medical Research Council guidance. BMJ.

[14] Wilson PM, Rodgers WM, Blanchard CM, Gessell J (2003). The relationship between psychological needs, Self-determined motivation, exercise attitudes, and physical fitness. Journal of Applied Social Psychology.

[15] Mullan E (1997). Markland D: Variations in self-determination across the stages of change for exercise in adults. Motivation and Emotion.

[16] Allender S, Cowburn G, Foster C (2006). Understanding participation in sport and physical activity among children and adults: a review of qualitative studies. Health Educ Res.

[17] Hagger MS, Chatzisarantis NL (2008). Self-determination theory and the psychology of exercise. International Review of Sport and Exercise Psychology.

[18] Carver CS, Scheier M (1998). On the self-regulation of behavior.

[19] Oettingen G, Gollwitzer PM, Forgas JP, Baumeister RF, Tice DM (2009). Making goal pursuits effective. Expectancy-dependent goal setting and goal striving. Psychology of Self-regulation: Cognitive, Affective, and Motivational Processes.

[20] Ryan RM, Deci EL (2000). Self-determination theory and the facilitation of intrinsic motivation, social development, and well-being. Am Psychol.

[21] Ryan RM, Patrick H, Deci EL (2008). Williams GC: Facilitating health behaviour change and its maintenance: Interventions based on Self-determination theory. The European Helath Psychologist.

[22] Deci EL, Ryan RM (2000). The what and why of goal pursuits: Human needs and the self-determination of behavior. Psychological Inquiry.

[23] Patrick H, Canevello A (2011). Methodological overview of a self-determination theory-based computerized intervention to promote leisure-time physical activity. Psychol Sport Exerc.

[24] Edmunds J, Ntoumanis N, Duda JL (2009). Helping your clients and patients take ownership over their exercise: Fostering exercise adoption, adherence, and associated well-being. Acsms Health & Fitness Journal.

[25] Reeve J (2009). Why teachers adopt a controlling motivating style toward students and how they can become more autonomy supportive. Educational Psychologist.

[26] Edmunds J, Ntoumanis N, Duda JL (2008). Testing a self-determination theory-based teaching style intervention in the exercise domain. European Journal of Social Psychology.

[27] Markland D, Ryan RM, Tobin VJ, Rollnick S (2005). Motivational interviewing and self-determination theory. Journal of Social and Clinical Psychology.

[28] Markland D, Vanstennkiste M, Hagger MS, Chatzisarantis NL (2007). Self-Determinarion theory and motivational interviewing in exercise. Intrinsic motivation and self-determination in exercise and sport.

[29] Crutzen R, de Nooijer J, Brouwer W, Oenema A, Brug J, de Vries NK (2008). Qualitative assessment of adolescents' views about improving exposure to Internet-delivers interventions. Health Education.

[30] Ridley K, Ainsworth BE, Olds TS (2008). Development of a compendium of energy expenditures for youth. Int J Behav Nutr Phys Act.

[31] Ainsworth BE, Haskell WL, Herrmann SD, Meckes N, Bassett DR, Tudor-Locke C, Greer JL, Vezina J, Whitt-Glover MC, Leon AS (2011). 2011 Compendium of Physical Activities: a second update of codes and MET values. Med Sci Sports Exerc.

[32] Pate RR, Pratt M, Blair SN, Haskell WL, Macera CA, Bouchard C, Buchner D, Ettinger W, Heath GW, King AC (1995). Physical activity and public health. A recommendation from the Centers for Disease Control and Prevention and the American College of Sports Medicine. JAMA.

[33] Michie S, Abraham C, Whittington C, McAteer J, Gupta S (2009). Effective techniques in healthy eating and physical activity interventions: a meta-regression. Health Psychol.

[34] Brouwer W, Kroeze W, Crutzen R, de Nooijer J, de Vries NK, Brug J, Oenema A (2011). Which intervention characteristics are related to more exposure to internet-delivered healthy lifestyle promotion interventions? A systematic review. J Med Internet Res.

[35] Crutzen R, de Nooijer J, Brouwer W, Oenema A, Brug J, de Vries NK (2011). Strategies to facilitate exposure to internet-delivered health behavior change interventions aimed at adolescents or young adults: a systematic review. Health Educ Behav.

[36] Kushniruk A (2002). Evaluation in the design of health information systems: application of approaches emerging from usability engineering. Comput Biol Med.

[37] Currie SL, McGrath PJ, Day V (2010). Development and usability of an online CBT program for symptoms of moderate depression, anxiety, and stress in post-secondary students. Computers in Human Behavior.

[38] Thompson D, Cullen KW, Boushey C, Konzelmann K (2012). Design of a website on nutrition and physical activity for adolescents: results from formative research. J Med Internet Res.

[39] Kushniruk AW, Patel VL, Cimino JJ (1997). Usability testing in medical informatics: cognitive approaches to evaluation of information systems and user interfaces. Proc AMIA Annu Fall Symp.

[40] (2004). Morville, P.

[41] Cohen L, Manion L, Morrison K (2007). Research methods in education.

[42] Johnson B (1997). Examining the validity structure of qualitative research. Education.

[43] Neuendorf KA (2002). The content analysis guidebook.

[44] Derks D, Agneta HF, Bos AER (2008). The role of emotion in computer-mediated communication: A review. Computers in Human Behavior.

[45] Walther JB (2001). D'Addario KP: The impacts of emoticons on message interpretation in computer-mediated communication. Social Science Computer Review.

[46] Morkes J Concise, scannable and objective: How to write for the web.

[47] Deforche BI, De Bourdeaudhuij IM, Tanghe AP (2006). Attitude toward physical activity in normal-weight, overweight and obese adolescents. J Adolesc Health.

[48] The Norwegian Directorate of Health (2009). Physical activity among children and young people in Norway.

